# Regression applied to protein binding site prediction and comparison with classification

**DOI:** 10.1186/1471-2105-10-276

**Published:** 2009-09-03

**Authors:** Joachim Giard, Jérôme Ambroise, Jean-Luc Gala, Benoît Macq

**Affiliations:** 1 Communications and Remote Sensing Laboratory, Université Catholique de Louvain, Place du Levant 2,1348 Louvain-la-Neuve, Belgium; 2 Center of Applied Molecular Technology, Université Catholique de Louvain, Clos Chapelle-aux-Champs 30, 1200 Bruxelles, Belgium

## Abstract

**Background:**

The structural genomics centers provide hundreds of protein structures of unknown function. Therefore, developing methods enabling the determination of a protein function automatically is imperative. The determination of a protein function can be achieved by studying the network of its physical interactions. In this context, identifying a potential binding site between proteins is of primary interest. In the literature, methods for predicting a potential binding site location generally are based on classification tools. The aim of this paper is to show that regression tools are more efficient than classification tools for patches based binding site predictors. For this purpose, we developed a patches based binding site localization method usable with either regression or classification tools.

**Results:**

We compared predictive performances of regression tools with performances of machine learning classifiers. Using leave-one-out cross-validation, we showed that regression tools provide better predictions than classification ones. Among regression tools, Multilayer Perceptron ranked highest in the quality of predictions. We compared also the predictive performance of our patches based method using Multilayer Perceptron with the performance of three other methods usable through a web server. Our method performed similarly to the other methods.

**Conclusion:**

Regression is more efficient than classification when applied to our binding site localization method. When it is possible, using regression instead of classification for other existing binding site predictors will probably improve results. Furthermore, the method presented in this work is flexible because the size of the predicted binding site is adjustable. This adaptability is useful when either false positive or negative rates have to be limited.

## Background

Structural genomics [1] is an important field, the objective of which is the determination of the three-dimensional structures of all the proteins coded by a genome. Recent advances in this field increase our understanding of protein function [2] but have also an impact on the pharmaceutical industry [3,4]. The structural genomics centers provide hundreds of protein structures of unknown function [5].

Development of automated protein function predictor from the structure is imperative and is nowadays an active research field in bioinformatics. One of the various approaches for assigning a function to a protein is to study the network of its physical interactions [6,7]. In this context, identifying a potential binding site between proteins is of primary interest. The localization of such binding site can also reduce the search space required by protein docking algorithms to predict the best match between two proteins. This localization has also an importance in studies about interactions between proteins and small molecules, such as ligands or substrates.

Bartlett et al. [8] presented an analysis of the properties of the catalytic residues. They showed that catalytic residues share common properties including the propensity of residue types, the conservation and the solvent accessibility. Petrova and Wu [9] compared 26 machine learning classifiers to specifically identify the catalytic residues within the whole enzyme. Gutteridge et al. [10] trained a neural network classifier to score the residues of a protein by their likelihood to be catalytic. The location of the active site were determined by searching for clusters of high-ranking residues.

Keskin et al., in their algorithm PRISM [11], used spatial similarity to predict protein-protein binding sites locations. Shulman-Peleg et al. proposed a method, called SiteEngine [12,13], comparing the properties at different regions of the proteins surface, such as conformational properties, to find proteins with similar functions in databases. The same kind of approach has been done by Shatsky et al. [14] to recognize binding patterns common to a set of protein structures. Jones and Thornton [15] presented a method to predict protein-protein interaction sites. The prediction was based on the calculation of relative combined scores for patches constructed on the surface of protein structures. Zhou and Shan [16] published a method based on a neural network for predicting individual residues in protein-protein interfaces. Since then, a lot of methods predicting individual residues or patches that overlap with interface have been proposed [9,17-22].

Bradford et al. [18] proposed a protein binding site predictor based on a Support Vector Machine (SVM) discriminating the patches constructed by segmentation of the surface. After the segmentation and the computation of some relevant properties for each patch of each protein, the SVM was trained and became usable to distinguish binding site from non-binding site surface parts. Bradford and Westhead [23] used a Bayesian network in combination with a surface patch analysis to design a protein-protein binding site predictor. Petsalaki et al. [24] presented a different approach to predict peptide binding sites on protein surface. This approach is based on the construction of spatial position specific scoring matrices for each of the 20 standard amino acid. Several groups performed the comparison of different tools usable for the prediction of protein-protein binding sites [25,26].

In the literature, patches based binding site localization methods generally use supervised classifier to find the relationship between the patches properties and their overlap with the true binding site. Machine learning or statistical techniques are used to discriminate patches totally superposed with the real binding site from patches lacking joined surface with it. In these cases, the response variable is categorical because it only takes two distinct values. So, supervised binary classifiers are used to predict the response variable from the predictor variables. However, when predicting the location of the binding site of a protein, a lot of patches constructed are partially superposed with the real binding site. Using regression tools in place of classification tools allows to include partially superposed patches during the training process what should improve the predictive performance of the model.

The first contribution of this paper is the description of a patches based protein-protein binding site localization method including either regression or classification tools. The predictive performance of several regression and classification tools are compared using leave-one-out cross-validation. Our method is also compared with three methods usable through Web Servers. The first method, named SHARP^2 ^[19], is a patches based binding site localization method using a combined scoring function. The second method, named PINUP [20], is based on an empirical scoring function including a side-chain energy term, a solvent accessibility term and a conservation term. The third method named cons-PPISP [17] is based on a neural network taking PSI-blast sequence profile and solvent accessibility as input. Another contribution is to consider the travel depth as one of the surface properties. The travel depth is a notion introduced by Coleman and Sharp [27] to quantify the depth of pockets within a molecular surface. The third contribution of our method is the construction of the predicted binding site. In the literature, for patches based methods, the predicted binding site in chosen among the original patches according to the ranking of their probability to be the real binding site. Our method takes into account the output of the statistical model on the whole surface to construct a new patch, which forms the predicted binding site. The size of this predicted binding site can be adjusted according to the application. Whereas a too small predicted binding site increases the risk of false negative results, a too large counterpart increases the risk of false positive results. Limiting false negative results avoids to miss a binding site even if the predicted zone is too large, whereas limiting false positive results ensures that predicted zones is a part of the real binding site.

## Methods

The aim of this work is to develop a method enabling to predict the location of potential binding sites in a protein according to its 3D structure. The development of this method is composed of two successive parts: the training and the application.

Input data of the training part are the PDB files of several protein complexes. External surfaces of the proteins are constructed by using the 3D structures encoded in the PDB files. Some characteristics are extracted and mapped on these surfaces. Then, the surfaces are segmented into patches. The mean values of the properties and an index quantifying the overlap with the real binding site are computed for each patch. Finally, a statistical model is used to find a relationship between the patches properties and their overlap indices.

Input data of the application part are one or more PDB files. Patches are constructed in the same way as in the training part except that the overlap index with the real binding site cannot be computed. The location of the real binding site is indeed unknown. The statistical model previously trained is then used to allocate a score to each patch. Finally, a predicted binding site is located by using the score mapped on the protein surface.

To validate the method, the application part can be tested on a protein of known binding site location. In this case, the location is considered as unknown during the prediction of the binding site location. Comparing the predicted location of the binding site with the real one, is used to validate the performance of our method.

**Figure 1:**
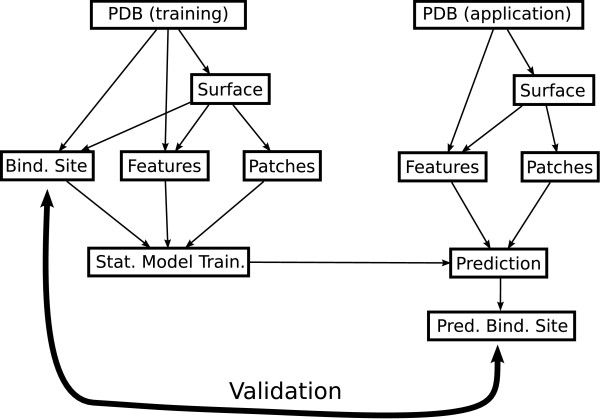
**Method pipeline overview**. Overview of the method pipeline. The input data are protein 3D conformations in PDB format. The surface of the proteins are computed and some properties are extracted. In the case of training proteins, the binding sites are identified. The surface are divided into patches which are associated with properties, and, in the case of training proteins, with the indices of the overlap with the binding site. The statistical model is trained and is used to give overlap scores to patches of application proteins. These scores are used to predict the locations of a potential binding sites.

A scheme of the method pipeline is depicted in Figure 1.

### PDB

The input data of the algorithm in both the training and application parts, are a set of protein 3D conformations. For the tests performed in this work, they are downloaded from the Protein Data Bank [28]. Two datasets are used in this work and both are composed of known protein complexes. The first one, used to train the models and to compare the statistical tools, is the one used by Bradford et al [18] (See additional file 1: Dataset1). This dataset is composed of proteins in their bounded conformation and had already been filtered at 20% sequence identity. To compare our method with existing methods, the dataset used consists of 35 proteins in the enzyme/inhibitor category of Docking Benchmark 2.0 [29], after filtering at 35% identity (See additional file 2: Dataset2). The genuine binding sites were identified using the bounded complexes but the tests were performed on the unbounded structures.

### Surface

**Figure 2:**
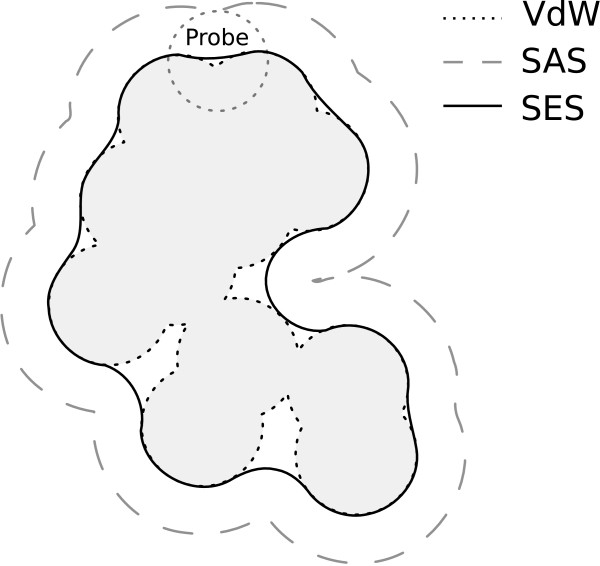
**Molecular surface representations**. Different ways to represent a molecular surface. The Van der Waals Surface (VdW: dots) is the exterior boundary of the union of Van der Waals spheres. The Solvent Accessible Surface (SAS: dashes) is the result of the VdW Surface dilatation by a structuring element, or a probe, representing a solvent molecule, typically water. The Solvent Excluded Surface (SES: solid line) is the results of the SAS erosion by the same probe.

There are different ways to represent a molecular surface (Figure 2) among which the Van der Waals surface, the Solvent Accessible Surface (SAS) and the Solvent Excluded Surface (SES) [30].

With the Van der Waals Surface Model, the electron clouds around atoms are approximated by rigid spheres of different radii, which are called the Van der Waals radii of the atoms. With the SAS Model, the inner surface of the volume is filled by the possible positions of the center of a ball representing a molecule of solvent (e.g., water). The SES Model is comparable but considering the exterior surface of the ball. In this work, we used the SES Model, which is approximated by a 3D mesh. A mesh is a collection of points, edges and faces defining surfaces in a 3D environment. As no conformation changes are taken into account in our method, proteins are considered to be rigid objects. It allows to work with surface points without considering what occurs inside the protein.

### Binding site

A residue is considered to lie in the binding site if more than 1 Å of its SES area is hidden after a complex formation [31]. As the proteins of the training data set originate from PDB files containing proteins complexes, the locations of their binding sites are already known.

### Properties

Properties of different types are affected to mesh points. These properties can be classified into three groups: geometric properties, composition and conservation score. In order to make the method more uniform, all properties are considered to be surface points properties. So, each point of the SES is related to the closest atom center and inherits its properties.

#### Geometric properties

Two geometric properties are considered: the local curvature and the travel depth.

**Figure 3:**
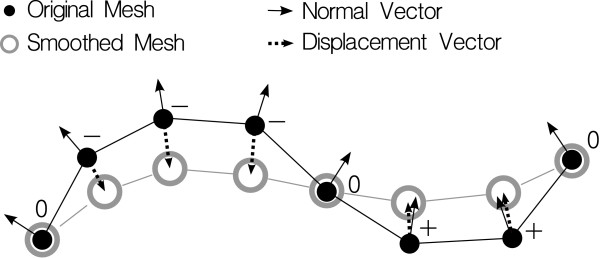
**Curvature estimation**. Scheme of the curvature estimation computation. The original mesh (black disks and links) is smoothed by a Laplacian operator to give a smoothed mesh (gray circles and links). The displacement vectors between both meshes are depicted with thick dotted arrows. The dot products between these vectors and the normal vectors of the original mesh (thin arrows) are calculated. The signs of the results are shown next to the original mesh vertices.

The mean curvature is negative for hollows, positive for bumps and zero for saddle points and planes. It is approximated as follows: first, the mesh is smoothed by a Laplacian operator and the displacement vector between each point position and the new position is evaluated. Then, the dot product between the point normal vector and the displacement vector is calculated. The curvature estimation is the normalized value of this product (Figure 3).

The travel depth of a surface point is defined as the shortest distance that a solvent molecule should do to reach this point from the convex hull of the SES. It is more global than the curvature computation while keeping a good surface resolution. It is computed with a surface-based algorithm using octrees [32].

#### Composition

The composition is the proportion of each surface amino acid composing a surface patch. For instance, if a fifth of the points in a patch corresponds to a leucine and the rest to a proline, the composition of the patch is Leu: 0.2, Pro: 0.8, other amino acids: 0. Other physico-chemical properties are redundant with the composition and are not significant for the statistical model.

#### Conservation score

The conservation score is a property in relation with the structure conservation across the evolution process. Key amino acid positions are often under strong evolutionary constraints. They are important for maintaining the 3D structure of a protein and/or its functions. Thus, the biological importance of a residue is often correlated with its level of evolutionary conservation within the protein family. The web-based tool Consurf  was used to calculate the evolutionary conservation score of each residue [33].

### Patches

Once the mesh is created and properties are affected to each point, patches are constructed. The construction is very simple. Patches are geodesic circles centered on each surface amino acid. The area of these circles is constant and proportional to the area of the whole SES. They are wider than the amino acid SES areas so that an important overlap exists between patches. The size of the patches is set empirically to one tenth of the whole SES and in this case, each surface point belongs to about fifteen patches. The mean of the properties are computed for each patches. Every patch in the training pipeline is also associated with an overlap index, the Positive Predicting Value (*PPV*), reflecting its similarity with the true binding site:



where *S *is the area of the SES, *P *is the analyzed patch and *I *is the true binding site. To give an intuitive vision of this measure, *PPV *is equal to one when the patch is completely included in the binding site. In the training part, the true binding site is also considered as an additional patch.

### Statistical tools

The objective of the statistical model is to assign a score to patches of a protein of unknown binding site location.

During the training part, the input data of the statistical model are the patches associated to their PPV and the properties extracted in the previous steps. The objective of the training is to find a relationship between the extracted patches properties (predictor variables), and the patches PPV (response variable). The relationship is used afterwards in the application part to allocate a score to the patches resulting from the segmentation of a target protein.

In this section, different statistical tools are presented. They can be separated into two categories: the classifiers, which are more commonly used to treat this kind of problems, and the regression tools. The output of a classifier is the probability of each patch to represent the real binding site. The output of a regression model is a predicted PPV for each patch. The statistical model output, whether it be a probability or a predicted PPV, is denominated the score of the patch. The score has to be highly correlated with the genuine PPV to ensure the success of the method.

#### Multiple linear regression (Lin. Reg.)

Multiple Linear Regression [34] is a statistical technique that fits the relationship between the response variable and the predictor variables, usually, by minimizing the sum of the squared deviations. The best multiple linear model can be found by using a stepwise selection of the predictor variables.

#### Partial least square regression (PLSR)

Partial Least Square Regression [35] is an extension of the multiple linear regression. Using multiple linear regression with a high number of predictor factors leads to over-fitting: the model fits the sampled data closely but fails to correctly predict new data responses. In a partial least square regression, a few latent factors are extracted and replace the original predictor factors for the response fitting. Partial least square is useful to find a good predictive model without necessarily understanding the existing relationship between variables.

#### Principal component regression (PCR)

Principal Component Regression [36] uses principal component analysis to fit the relationship between the predictor variables and the response variable. The first step is to compute the principal components of the predictor variables. The second step is the regression on a subset of the principal components.

#### Multilayer perceptron (MLP)

Multilayer Perceptron [37] is a machine learning technique using multiple layers of neurons. A neuron is a simple processing element, connected to the neurons of the previous and of the following layers. The connections between the neurons are characterized by weights which are adjusted during the training step. Multilayer Perceptrons can be used for regression as well as for supervised classification task. In this work, it was used as a regression tool.

#### Random forest for regression (RFR) and classification (RFC)

Random Forest [38] is a machine learning tool used for classification and regression tasks. In both cases, a random forest is a set of trees created by bootstrapping samples of the training data set. Random forests for regression use a set of regression trees. In this case, the prediction is made by averaging the predictions of the different trees. Random forests for classification use a set of classification trees. In this case, the prediction is made by a majority vote on the predictions of the different trees.

Regression trees and classification trees are both decision trees. A decision tree is a technique used to predict a response variable using a set of predictor variables. Regression trees are used for continuous response variables, whereas classification trees are used for discrete response variables. In both cases, it uses a series of *"if then else" *conditions based on values of the predictors to predict the response. During the training phase, the decision tree is built through an iterative process of data splitting. This iterative process continues until each node reaches a fixed minimum size. For instance, if there are three predictor variables, the data can be separated from the root according to the value of the first variable. At each new node, the data can then be separated according to the value of other variables, and then, at children nodes, they can be separated according to the first variable again, ... During the prediction phase, a new data starts from the root and follows the branches corresponding to the value of its predictor variables. The leaves determine the value of the response variable.

#### Naive bayes classifier (NBC)

Naive Bayes Classifier [39] is a machine learning tool using the bayes theorem to compute the probability of a case to belong to a category. The NBC is based on a conditional independence assumption. Given the value of the response variable, the predictor variables have to be independent. Despite the fact that this assumption is rarely true in reality, naive Bayes classifier often performs better than expected.

#### Support vector machine (SVM)

Support Vector Machine [40] is a machine learning tool used for classification and regression. When used for binary classification, the objective of the SVM is to map the training data into a property space by the aid of a kernel function, and to constructs an N-dimensional hyperplane that optimally separates the case according to the category of their response variable.

### Predicted binding site construction

After statistical analyses, the score assigned to each patch is used to construct a score map on the protein SES. Each point of the surface is associated to the geodesically closest center of patch and get the score affected to this patch. Next, the points of the mesh representing the SES are added successively to the predicted binding site in descending order of score. The growth of the predicted binding site stops when either a predetermined size or a score threshold is reached.

## Results and discussion

The two major consecutive estimation steps of our method are the statistical prediction and the predicted binding site construction. The predicted binding site construction is divided in two steps: the score mapping on the SES and the score map thresholding. Results comparing, on one hand, the different statistical tools, and, on the other, our method to other existing methods are presented in this section. The different statistical tools were compared after the statistical prediction by calculating the correlation coefficient between predictions and real PPVs. Next, they were compared after the mapping step by analyzing the score distribution inside and outside the genuine binding site. Finally, overlapping indices between the predicted and the real binding site were computed. These indices were used to compare the results obtained with different statistical models, as well as to compare results of our method with results of other existing methods.

### Statistical prediction

The performance of our method strongly depends on the ability of the statistical model to correctly classify a patch or to predict the corresponding PPV. For both classification and regression tools, the output is a number between 0 and 1 called the *score *of the patch.

Each classification and regression tool was evaluated through leave-one-out cross-validation, which is an iterated process. At each step, the data set, comprising the patches and their properties, was divided into two sub-sets: a test data set, which contained the patches of one protein, and a training data set, which contained the patches of all the other proteins. The statistical model was trained on the training data set, and used to affect a score to the patches of the test data set. The scores were finally compared to the real PPV through the computation of the Pearson Correlation Coefficient. The protein in the test data set was different at each iteration until prediction and correlation coefficient were obtained for the patches of each protein in the complete data set.

**Figure 4:**
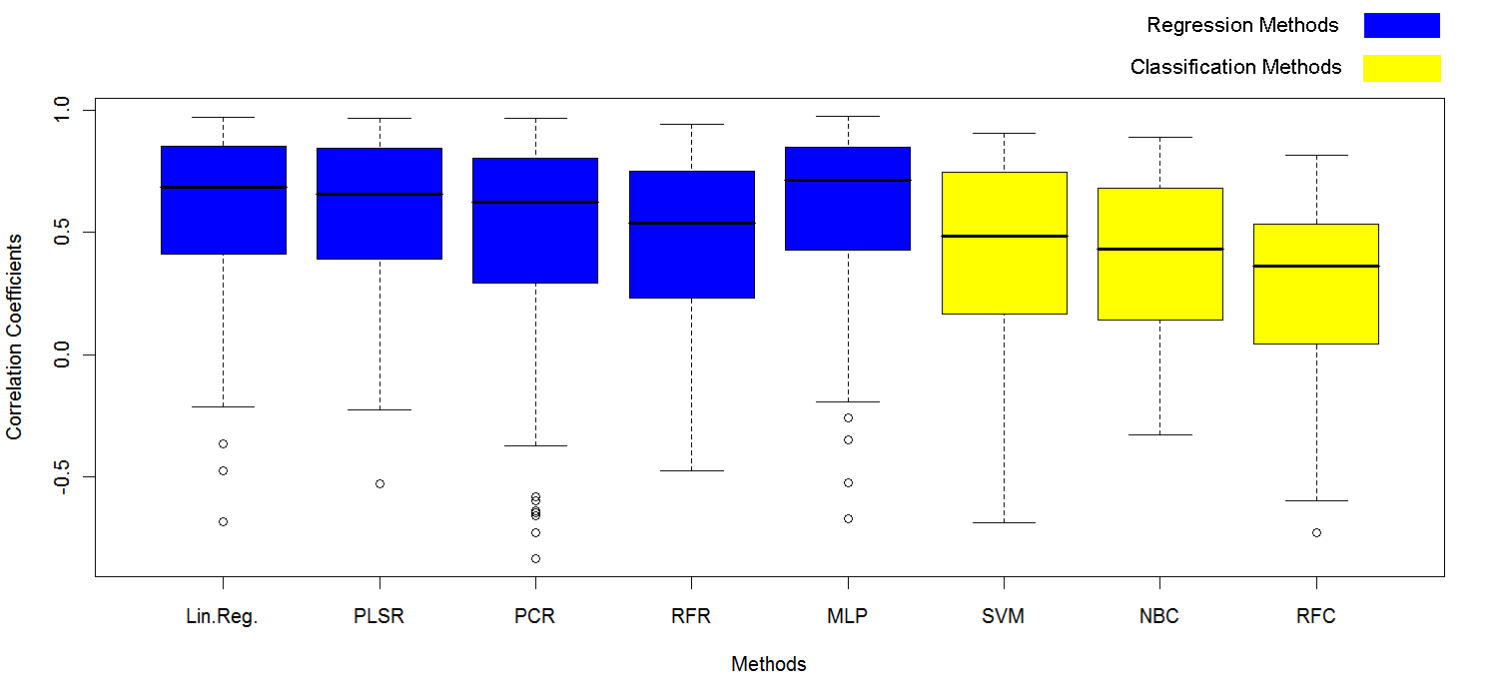
**Correlation coefficients box plot**. Box plot of the correlation coefficients for each statistical tool. Each correlation coefficient is computed between the scores of the patches of one protein and their real PPV. Each statistical tool is characterized by 180 correlation coefficients, corresponding to the 180 proteins used for the validation of our method. Coefficients for regressions are shown in blue and coefficients for supervised classification are shown in yellow.

**Figure 5:**
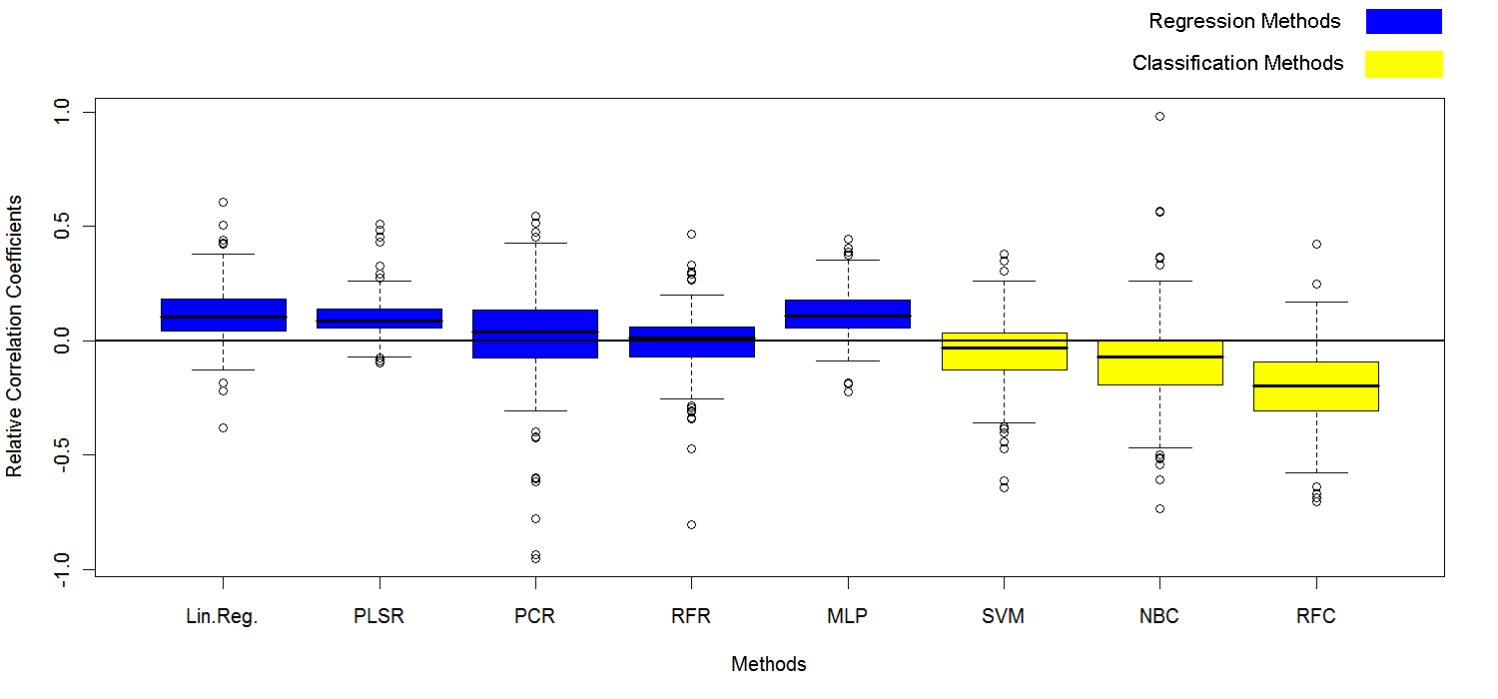
**Relative correlation coefficients box plot**. Box plot of the relative correlation coefficients for each statistical tool. The relative correlation coefficients are positive if the statistical tool gives better prediction than average. Coefficients for regressions are shown in blue and coefficients for supervised classification are shown in yellow.

After the leave-one-out cross-validation, each statistical tool was associated to 180 correlation coefficients, each of them corresponding to one protein. Distributions of these correlation coefficients for regressions and classifications are compared in the Box Plot shown in Figure 4. Using regression to fit the relationship between the properties and the PPV frequently led to better score correlations than using classification. As the correlation coefficients vary considerably from one protein to another for the same statistical tool, the box plots are stretched and overlap a lot. Building a relative box plot allows to better visualize which statistical tool generally gives the best results. For this purpose, relative correlation coefficients were computed for each protein separately, relatively to the mean of the scores for all the statistical tools. A positive relative coefficient means that the statistical tool better works than the average. The distribution of these relative coefficients is depicted in a box plot in Figure 5. The box plot of the relative correlation coefficients shows that, for one particular protein, regressions generally gave better prediction than the average, whereas classification generally gave worse prediction than the average.

### Binding site localization

After statistical prediction, a predicted binding site was constructed in two steps, namely the score map construction and the thresholding on the score map. The score map construction was validated by comparing the score distribution inside and outside the genuine binding site, using a histogram. The thresholding on the score map was validated by analyzing the PPV and the sensitivity of the resulting predicted binding site. The validation results for the different statistical tools are compared in this section.

#### Score map histograms

**Figure 6:**
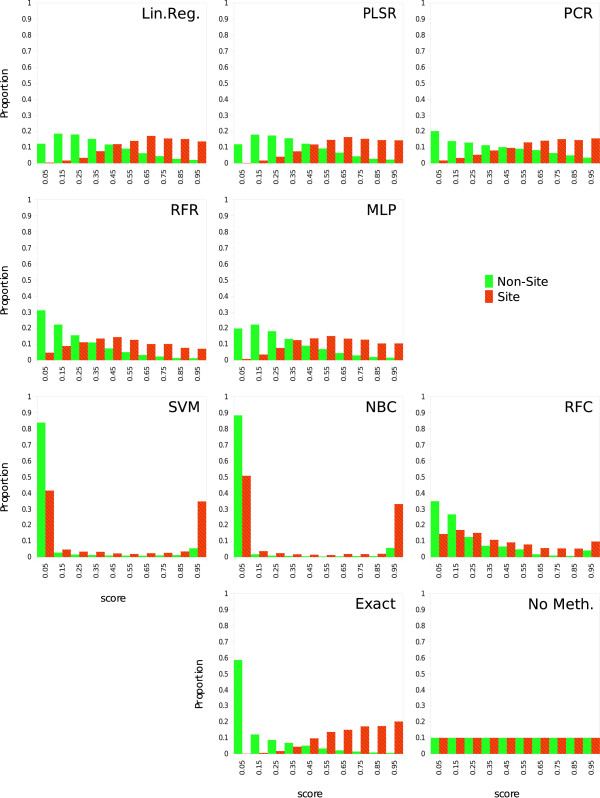
**Score map histograms**. Histograms of the score repartition inside the real binding site (red hatched bars) and on the rest of the molecular surface (green bars). The ideal histogram would be a single green bar on the far left side and a single red hatched bar on the far right side. The "Exact" histogram (lower center) is obtained by considering the real patches PPV as the score. The resulting error comes from the patches approximation only. The "No Meth." histogram (lower right) represents the expected histogram obtained if no binding site prediction method is used. Other histograms are for the different statistical tools.

For each statistical tool, the score distribution of the points within the real binding site was compared with the score distribution of the points within the rest of the protein surface. The score was weighted by the area of the targeted zones. Ten bins histograms are presented in Figure 6.

A histogram is given when the exact PPVs are taken as the score, another one represents the expected values for a random method, and each other one corresponds to a specific statistical tool. Red hatched bars represent the proportion of score for surface points belonging to the binding site and green plain bars for points belonging to the rest of the surface. An ideal histogram would show 100% of the observation in the first bin for the non-site parts and 100% of the observations in the last bin for the site parts. In the histograms corresponding to classifications (RFC, SVM, NBC), a lot of binding site parts have a small score, what leads to a lot of false negative results. On the other hand, in the histograms corresponding to regressions (Lin. Reg., RFR, MLP, PLSR, PCR), as for the "Exact" histogram, the repartition is more distinct between site and non-site zones, what makes the prediction more accurate.

#### Binding site prediction

**Figure 7:**
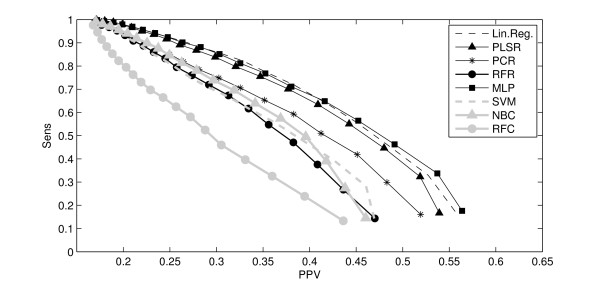
**Precision-recall curves for different statistical tools**. Precision-Recall Curves comparing the results obtained with different statistical models. The y-axis represents the mean sensitivity (or the precision) over the 180 proteins and the x-axis represents the mean PPV (or Recall). Curves corresponding to regression tools are depicted with thin black lines and curves corresponding to classification tools are depicted with thick gray lines.

The location of potential binding site is predicted by applying a threshold on the score map. Then, the results vary with the chosen threshold and can be adapted to limit the false positive or false negative rates. The predicted binding site was compared with the real one using two indices: the sensitivity (or Recall) and the Positive Predicted Value (or Precision). They are defined as follow:





where *S *is the area of the SES, *P *is the analyzed patch and *I *is the true binding site. Our whole method was tested using different statistical tools. A Precision-Recall graph comparing results for all these tools appears in Figure 7. Curves corresponding to regression tools generally are above the one corresponding to classification tools, what is consistent with the previous observations.

In this experiment, the size of the predicted binding site was arbitrarily set to 1/10 of the whole surface of the protein, i.e., points with the highest score were added to the predicted binding site until it attained 1/10 of the total surface. Results were compared to the expected indices obtained via random selection of the predicted binding site. As in this application, the area of the binding site and the area of the predicted binding site are unchanged for one protein, the expected values of both indices are estimated as follows:





where *S*_*T *_is the whole surface area. *S*(*I*) and *S*(*P*) are considered to be constant. So, in these formulas, the only variable is the overlap between *P *and *I*. The expected proportion of *P *inside *I *is the same as anywhere else on the surface. Then, *E *(*S*(*P *∩ *I*)) is this proportion weighted by *S*(*I*). The reasoning is the same for the proportion of *I *inside *P*. To quantify the success rate of our method with each statistical tool, the percentage of proteins of the data set with a higher index than expected (via random selection) was calculated (Table 1). As for other tests, the results are better for regressions than for classifications. In 84% of the cases, the MLP gave a better result than the expected value, whereas the best classification tool (NBC) gave a result of 78%.

**Table 1:** Success rate for different statistical tools.

**Statistical Tool**	**Success Rate**
Exact	100

Lin. Reg.	81.67

PLSR	82.78

PCR	76.67

RFR	77.78

MLP	83.89

SVM	75.00

NBC	77.78

RFC	69.44

Percentage of proteins in the whole data set resulting on a PPV higher than the expected value for each tool.

**Figure 8:**
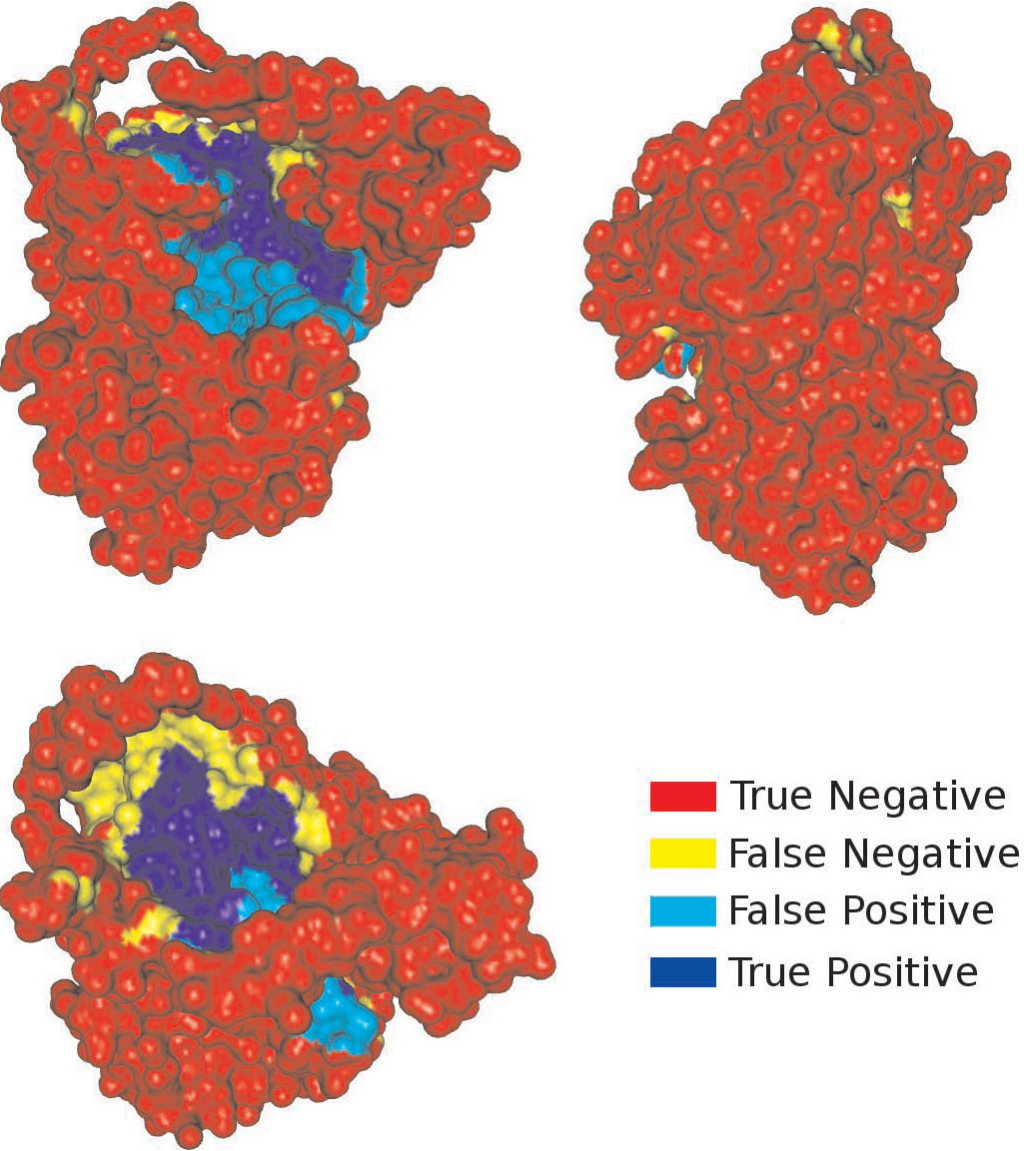
**Predicted binding site example**. Three views of protein SES with non labeled zones (red), zones belonging to the real binding site and not to the predicted one (yellow), zones belonging to the predicted binding site and not to the real one (light blue), and well predicted binding site zones (medium blue). To sum up, the predicted binding site is the union of the light blue and the medium blue zone, and the real binding site is the union of the yellow and the medium blue zones. Here, the predicted binding site has a sensitivity = 0.44 and a PPV = 0.54, which represent approximately the mean results using the MLP.

The tool giving the best results was the MLP with a mean sensitivity and PPV of 0.34 and 0.54, respectively. A molecule with a predicted binding site representing approximately these mean values is depicted in Figure 8.

#### Comparison with other methods

**Figure 9:**
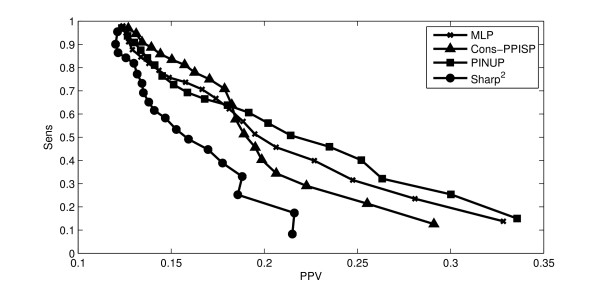
**Precision-recall curves for different methods**. Precision-Recall Curves comparing the results obtained with different models. The y-axis represents the mean sensitivity (or the precision) over the 180 proteins and the x-axis represents the mean PPV (or Recall). The MLP curve (line with crosses) is obtained using our method with a Multilayer Perceptron.

Finally, our method was compared to other methods for which applications are available on the web: Cons-PPISP [17], PINUP [20] and Sharp^2 ^[19]. These three methods return a score for each residue. These scores were mapped on the protein surfaces and the binding site localizations were predicted as it was done for the scores resulting from the different statistical models. The tests were performed on the second dataset (See Supplementary Materials 2). A Precision-Recall graph comparing results for all these methods appears in Figure 9. Performances of our method using MLP are higher than those of Sharp^2 ^method and comparable to those of the PINUP and Cons-PPISP methods. The percentage of proteins of the data set with a higher index than expected (via random selection) was also calculated (Table 2). For both our method using MLP and the PINUP method, the result was better than expected in 71% of the cases. These results are a bit worse than for the other dataset, probably because the training dataset was made of proteins from bounded structures.

**Table 2:** Success rate for different methods.

**Method**	**Success Rate**
MLP	71.43

Cons-PPISP	69.70

PINUP	71.43

Sharp^2^	62.86

Percentage of proteins in the whole data set resulting on a PPV higher than the expected value for each method.

## Conclusion

In this paper, we present a patch based binding site prediction method based on either classification or regression tools. This was motivated by the fact that patches based method presented in the literature generally use classification tools. In this case a binary classifier is trained to discriminate patches totally superposed with the real binding site from patches lacking joined surface with it. Using regression instead of classification allows to include patches partially overlapping the genuine binding site during the training step. The variable to be estimated by the regression is the overlap between the real binding site and the patches constructed on the protein surface.

Using leave-one-out cross-validation, we showed that regression tools have better predictive performance than classification ones. As the patches constructed during the application step partially overlap the genuine binding site, the predictions for these patches generally are more correlated with their PPV when regression is used. Among regression tools, the Multilayer Perceptron is the most efficient. In 84% of cases, with dataset 1 (See Supplementary Materials), the method using an MLP for regression, allowed a better prediction than the expected value via random selection. Our method used with MLP was also compared with three methods usable through a web server. Our method performed better than Sharp^2^, which is also a patches based method, and performed equivalently to the two other methods.

To sum up, regression tools appeared to be more efficient than classification tools for a new patches based method comparable with existing binding site prediction methods. When possible, using regression instead of classification for other predictors will probably improve the results, not only when patches are used, but every time the output is a continuous variable.

The method presented in this paper is also flexible. Indeed, the final predicted binding site is obtained by applying a threshold on the surface score map derived form the prediction of the regression or the classification tools. So, the size of the final predicted binding site is adaptable to the approximate false positive and negative rates required for the final application. Therefore, the output of our method is a single predicted binding site instead of a list of top ranked patches [18,23] or residues [9].

In future works, our method will be applied on real cases and combined with other bioinformatics tools. For instance, the combination of our method with dynamic molecular simulation will be used to study the impact of residue mutation on the location of the binding site.

## List of abbreviations

PDB: Protein Data Bank; SES: Solvent Excluded Surface; SAS: Solvent Accessible Surface; PPV: Positive Predictive Value; Lin.Reg.: Multiple Linear Regression; SVM: Support Vector Machine; PLSR: Partial Least Square Regression; PCR: Principal Component Regression; MLP: Multilayer Perceptron; RFR: Random Forest Regression; RFC: Random Forest Classifier; NBC: Naive Bayes Classifier.

## Authors' contributions

JG developed the features extraction part, the patch construction, the predicted binding site localization. He participated at the whole writing process. JA applied and tested the statistical tools. He participated at the whole writing process. JLG supervised the biological part of the work. He helped to finalize the manuscript. BM supervised the whole work. He helped to finalize the manuscript. All authors read and approved the final manuscript.

## Supplementary Material

Additional file 1 **Dataset1**. Dataset1.pdf contains the list of the 180 proteins pdb codes and names of the first dataset. It is the dataset used by Bradford et al. [18]. In this work, it was used for the statistical models training and for the comparison between the different statistical tools. This dataset is composed of proteins in the bounded conformation and had already been filtered at 20% sequence identity. Click here for file

Additional file 2 **Dataset2**. Dataset2.pdf describes the second dataset. It consists of 35 proteins in the enzyme/inhibitor category of Docking Benchmark 2.0 [29], after filtering at 35% identity. The genuine binding site was identified using the bounded complex but the tests were performed on the unbounded structures. The first column of the file contains the pdb codes of the bounded complexed structures. The second and the third column contain the pdb codes and the names of the unbounded structures of the enzymes and inhibitors respectively. Click here for file
