# Supplementary material for: Regression applied to protein binding site prediction and comparison with classification
Source: BMC Bioinformatics. 2009 Sep 3;10:276. doi: 10.1186/1471-2105-10-276 (PMC2749839; doi:10.1186/1471-2105-10-276)
Supplement: Additional file 1 — Dataset1. Dataset1.pdf contains the list of the 180 proteins pdb codes and names of the first dataset. It is the dataset used by Bradford et al. [18]. In this work, it was used for the statistical models training and for the comparison between the different statistical tools. This dataset is composed of proteins in the bounded conformation and had already been filtered at 20% sequence identity. [file 1471-2105-10-276-S1.pdf]

Dataset 1 (Bradfor et al.)

Protein chains involved in non-obligate interactions

Enzyme-inhibitors:

1a4y_A ribonuclease inhibitor
1a4y_B angiogenin
1ava_A alpha amylase
1avw_A trypsin
1avw_B soybean trypsin inhibitor
1ay7_A ribonuclease Sa
1ay7_B Barstar
1bvn_T tendamistat
1clv_I alpha amylase inhibitor
1cse_I eglin C
1dpj_A proteinase A
1dpj_B proteinase inhibitor Ia3
1dtd_A carboxypeptidase A2
1dtd_B metallocarboxypeptidase inhibitor
1eai_C elastase/chymostrypsin inhibitor I
1f34_B major pepsin inhibitor
1fss_A acetylcholinesterase
1fss_B fasciculin II
1gla_F Glucose-Specific Factor III
1kxq_H antibody Vhh fragment
1mct_I squash seed inhibitor
1smp_A serratia metalloproteinase
1smp_I erwinia chysathemi inhibitor
1tab_I bowman-birk inhibitor
1tgs_I pancreatic secretory trypsin inhibitor
1udi_E uracil-DNA glycosylase
1udi_I UDG inhibitor
1viw_B alpha amylase inhibitor
2ptc_I bovine pancreatic trypsin inhibitor
2sic_E subtilisin BPN
2sic_I SSI
4cpa_I potato carboxypeptidase A inhibitor
4sgb_E serine proteinase B
4sgb_I potato chymotrypsin inhibitor I
7cei_A Im7
7cei_B DNAase

Others:

1agr_E Rgs 4
1atn_D Deoxyribonuclease I
1b6c_A Fk506-Binding Protein
1b6c_B Tgf-B Superfamily Receptor Type I
1bkd_S Son Of Sevenless-1
1buh_B Cell cycle regulatory protein Ckshs1
1d2z_B Death Domain Of Tube
1dow_A alpha-catenin
1dow_B beta-catenin A
1eay_A chey
1eay_C chea
1euv_A Ulp1 Protease
1euv_B Ubitquitin-Like Protein Smt3
1f3v_B traf2 2.0A
1f5q_A Cyclin Dependent Kinase 2
1f5q_B Cyclin
1he1_A Exoenzyme S
1hx1_B Bag-Family Molecular Chaperone Regulator-1
1i2m_B Regulator Of Chromosome Condensation
1i8l_C Cytotoxic T-Lymphocyte Protein 4
1kac_B Adenovirus Receptor
1pdk_A Chaperone Protein Papd
1pdk_B Protein Papk
1qav_A alpha-1 Syntrophin
1tx4_A P50-Rhogap
1tx4_B Rhoa
1xdt_R Heparin-Binding Epidermal Growth Factor
1xdt_T Diphtheria Toxin
3ygs_C Apoptotic Protease Activating Factor
3ygs_P Procaspase 9

Protein chains involved in obligate interactions

Hetero-dimers:

1ahj_A nitrite hydratase
1ahj_B nitrite hydratase
1aht_H alpha thrombin
1aht_L alpha thrombin
1b34_A Small Nuclear Ribonucleoprotein Sm 2
1b34_B Small Nuclear Ribonucleoprotein Sm 2
1bun_A beta-2-Bungarotoxin
1dce_A Rab Geranylgeranyltransferase

1dce_B Rab Geranylgeranyltransferase
1dj7_A ferredoxin thioredoxin
1efv_A electron transfer flavoprotein
1efv_B electron transfer flavoprotein
1g4y_B calcium-activated potassium channel Rsk
1g4y_R calmodulin
1gux_A Retinoblastoma Protein
1gux_B Retinoblastoma Protein
1h2a_L Ferricytochrome-C3 Oxidoreductase
1h2a_S Ferricytochrome-C3 Oxidoreductase
1luc_B Bacterial Luciferase
1pnk_A Penicillin Amidohydrolase
1pnk_B Penicillin Amidohydrolase
1req_A Methylmalonyl-Coa Mutase
1req_B Methylmalonyl-Coa Mutase
1tco_A serine/threonine phosphatase 2B
1tco_B serine/threonine phosphatase 2B
2aai_A ricin
2aai_B ricin

Homo-dimers:

1a0f_B Glutathione S-Transferase
1a4i_A Methylenetetrahydrofolate Dehydrogenase
1a4u_A Alcohol Dehydrogenase
1afr_F delta-9 Stearoyl-Acyl Carrier Protein
1afw_A 3-Ketoacetyl-Coa Thiolase
1aj8_A Citrate Synthase
1ajs_A Aspartate Aminotransferase
1aom_A Nitrite Reductase
1aq6_A L-2-Haloacid Dehalogenase
1at3_A Herpes Simplex Virus Type II Protease
1az3_B Ecorv Endonuclease
1b3a_B Rantes
1b5e_A Deoxycytidylate Hydroxymethylase
1b7b_A Carbamate Kinase
1b8a_A Aspartyl-tRNA Synthetase
1b8j_B Alkaline Phosphatase
1b9m_B Molybdate-Dependent Transcriptional Regulator
1bbh_A cytochrome C
1bft_A Nuclear Factor Nf-kappa-B P65
1bjn_B Phosphoserine Aminotransferase
1bo1_A Phosphatidylinositol Phosphate Kinase
1brm_A Aspartate-Semialdehyde Dehydrogenase

1bw0_B Tyrosine Aminotransferase
1byf_A Polyandrocarpa lectin
1byk_B Trehalose Operon Repressor
1c7n_A Cystalysin
1cli_B Phosphoribosyl-Aminoimidazole Synthetase
1cmb_B Met Apo-Repressor (Metj)
1cnz_A 3-Isopropylmalate Dehydrogenase
1coz_A Glycerol-3-Phosphate Cytidylyltransferase
1cp2_A Nitrogenase Iron Protein
1dor_A Dihydroorotate Dehydrogenase A
1e0b_A Swi6 Protein
1ete_A Flt3 Ligand
1f5m_A Gaf
1f6y_A Iron Sulfur Protein Methyltransferase
1f8r_A L-Amino Acid Oxidase
1gpe_B Glucose Oxidase
1hgx_B Hypoxanthine-Guanine-Xanthine Phosphoribosyltransferase
1hjr_C RuvC resolvase
1hss_A 0.19 alpha-Amylase Inhibitor
1hul_A Interleukin-5
1isa_B Iron(II) Superoxide Dismutase
1jkm_A Brefeldin A Esterase
1kpe_A Protein Kinase C Interacting Protein
1mka_A beta-Hydroxydecanoyl Thiol Ester Dehydrogenase
1msp_A Major Sperm Protein
1nse_A Nitric Oxide Synthase
1nsy_A Nad Synthetase
1one_A Enolase
1pp2_L Phospholipase A2
1pvu_A Pvuii Restriction Endonuclease
1qae_B Extracellular Endonuclease
1qax_A 3-Hydroxy-3-Methylglutaryl-Coenzyme
1qbi_A Soluble Quinoprotein Glucose Dehydrogenase
1qfe_A 3-Dehydroquinate Dehydratase
1qfh_B Actin Binding Protein 120
1qi9_B Vanadium Bromoperoxidase
1qor_B Quinone Oxidoreductase
1qqj_A Fumarylacetoacetate Hydrolase
1qu7_B Methyl-Accepting Chemotaxis Protein
1scf_B Stem Cell Factor
1smt_A Transcriptional Repressor Smtb
1sox_A Sulfite Oxidase
1spu_A Copper Amine Oxidase
1trk_B Transketolase

Dataset 1 (Bradfor et al.)

1vfr_B Nad(P)H: Fmn Oxidoreductase
1vhi_A Epstein Barr Virus Nuclear Antigen-1
1vlt_A Aspartate Receptor
1vok_A Tata-Box-Binding Protein
1vsg_B Variant Surface Glycoprotein
1wgj_A Inorganic Pyrophosphatase
1xik_A Protein R2 Of Ribonucleotide Reductase
1xso_A Cu, Zn Superoxide Dismutase
1ypi_A Triose Phosphate Isomerase (TIM)
1yve_J Acetohydroxy Acid Isomeroreductase
2ae2_A Tropinone Reductase-II
2arc_A Arabinose Operon Regulatory Protein
2gsa_B Glutamate Semialdehyde Aminotransferase
2hdh_A L-3-Hydroxyacyl Coa Dehydrogenase
2hhm_B Inositol Monophosphatase
2nac_B Formate Dehydrogenase
2pfl_B Pyruvate Formate-Lyase
2utg_B Uteroglobin
3tmk_B Thymidylate Kinase
4mdh_A Cytoplasmic Malate Dehydrogenase
5hvp_B HIV-1 Protease
