# Supplementary material for: Regression applied to protein binding site prediction and comparison with classification
Source: BMC Bioinformatics. 2009 Sep 3;10:276. doi: 10.1186/1471-2105-10-276 (PMC2749839; doi:10.1186/1471-2105-10-276)
Supplement: Additional file 2 — Dataset2. Dataset2.pdf describes the second dataset. It consists of 35 proteins in the enzyme/inhibitor category of Docking Benchmark 2.0 [29], after filtering at 35% identity. The genuine binding site was identified using the bounded complex but the tests were performed on the unbounded structures. The first column of the file contains the pdb codes of the bounded complexed structures. The second and the third column contain the pdb codes and the names of the unbounded structures of the enzymes and inhibitors respectively. [file 1471-2105-10-276-S2.pdf]

Dataset 2 (Docking Benchmark 2.0)

Complex	Enzyme	Inhibitor
1AVX_A:B		1BA7_B Soybean trypsin inhibitor
1AY7_A:B	1RGH_B Barnase	1A19_B Barstar
1BVN_P:T		1HOE_ Tendamistat
1CGI_E:I		1HPT_ PSTI
1D6R_A:I		1K9B_A Bowman-Birk inhibitor
1DFJ_E:I	9RSA_B Ribonuclease A	2BNH_ Rnase inhibitor ACE
1E6E_A:B	1E1N_A Adrenoxin reductase FAD	1CJE_D Adrenoxin
1EAW_A:B	1EAX_A Matriptase	9PTI_ BPTI
1EWY_A:C	1GJR_A Ferredoxin reductase FAD	1CZP_A Ferredoxin
1EZU_C:AB		1ECZ_AB Ecotin
1F34_A:B	4PEP_ Porcine pepsin	1F32_A Ascaris inhibitor
1HIA_AB:I	2PKA_XY Kallikrein	1BX8_ Hirustatin
1MAH_A:F	1J06_B Acetylcholinesterase	1FSC_ Fasciculin
1TMQ_A:B	1JAE_ alpha-amylase 5HP	1B1U_A RAGI inhibitor
1UDI_E:I	1UDH_ Uracyl-DNA glycosylase	2UGI_B Glycosylase inhibitor
2MTA_HL:A	2BBK_JM Methylamine dehydrogenase	2RAC_A Amicyanin
2PCC_A:B	1CCP_ Cyt C peroxidase HEM	1YCC_ Cytochrome C HEM
2SIC_E:I		3SSI_ Streptomyces subtilisin inhibitor
2SNI_E:I	1UBN_A Subtilisin SOC	2CI2_I Chymotrypsin inhibitor
7CEI_A:B	1UNK_D Colicin E7 nuclease	1M08_B Im7 immunity protein
